# Thyrotoxic Atrial Fibrillation: Factors Associated with Persistence and Risk of Ischemic Stroke

**DOI:** 10.1155/2017/4259183

**Published:** 2017-12-12

**Authors:** Cheuk-Lik Wong, Ho-Kee Vicki Tam, Chun-Kit Vincent Fok, Pong-Kai Ellen Lam, Lai-Ming Fung

**Affiliations:** Department of Medicine & Geriatrics, Caritas Medical Centre, 111 Wing Hong Street, Sham Shui Po, Kowloon, Hong Kong

## Abstract

**Background:**

Atrial fibrillation (AF) is one of the commonest cardiovascular manifestations of thyrotoxicosis. A significant proportion of patients have persistent AF which may have long term consequences, for example, ischemic stroke.

**Methods:**

We performed a retrospective cohort study in a regional hospital from January 2004 to June 2016 to examine the clinical characteristics and outcomes of thyrotoxic patients who presented with atrial fibrillation and to investigate possible factors associated with persistent atrial fibrillation and ischemic stoke.

**Results:**

Among 1918 patients who had a diagnosis of thyrotoxicosis, 133 (6.9%) patients presented with AF. Spontaneous sinus conversion occurred in 89 (66.9%) patients in which 85 (94%) patients developed sinus conversion before or within 6 months after having achieved euthyroidism. The remaining 44 (33.1%) had persistent AF. The rate of ischemic stroke was numerically higher among patients who had persistent AF than those with spontaneous sinus conversion (15.9% versus 10.1%; log-rank 0.442, *p* = 0.506). Patients who sustained an ischemic stroke were older (71 ± 11 years versus 62 ± 16 years, *p* = 0.023) and had a trend towards higher CHA_2_DS_2_-VASc score (2.9 ± 1.7 versus 2.3 ± 1.7, *p* = 0.153). History of smoking (adjusted odds ratio 4.9, 95% CI [1.8,14.0], *p* = 0.002), a larger left atrial diameter (adjusted odd ratio 2.6, 95% CI [1.2,5.5], *p* = 0.014), and a relatively lower free thyroxine level at diagnosis (adjusted odd ratio 2.1, 95% CI [1.2,3.5], *p* = 0.008) were associated with persistence of AF on multivariate analysis.

**Conclusion:**

Persistence of thyrotoxic AF occurred in one-third of patients and spontaneous sinus conversion was unlikely after six months of euthyroidism. High rate of ischemic stroke was observed among patients with persistent thyrotoxic AF and older age. Patients with factors associated with persistent AF, especially older people, should be closely monitored beyond 6 months so that anticoagulation can be initiated in a timely manner to reduce risk of ischemic stroke.

## 1. Introduction

Thyroid hormones have multiple effects on the cardiovascular system and play a fundamental role in cardiovascular homeostasis in both physiological and pathological conditions. The heart is particularly sensitive to the change in local tri-iodothyronine (T3) levels as T3 is essential in maintaining cardiac morphology and function [1]. Hyperthyroidism is associated with significant changes in the cardiovascular system including increase in heart rate, venous return, and stroke volume resulting in increased cardiac output, reduced peripheral vascular resistance, and increased atrial automaticity [2–6]. Patients with hyperthyroidism often manifest prominent cardiovascular symptoms and signs and a significant proportion of them develop specific cardiovascular complications including atrial fibrillation, heart failure, pulmonary hypertension, and dilated cardiomyopathy as well as ventricular arrhythmias.

Patients with overt hyperthyroidism are at risk of increased mortality. Cardiovascular disease has been implicated as the major cause for the excess mortality in a number of observational studies [7]. In a meta-analysis by Brandt et al., the mortality of subjects with overt hyperthyroidism was increased by 20% compared with the controls although there was pronounced heterogeneity in the studies included [8]. In a population-based cohort study in Finland, the rate of hospitalisation due to cardiovascular disease, including heart failure, atrial fibrillation, hypertension, and cerebrovascular disease, was higher among patients with hyperthyroidism than among the controls [9]. Similarly, the same group and another US group also reported an excess risk of cardiovascular mortality among subjects with hyperthyroidism compared to the general population even after treatment with radioactive iodine. The excess mortality was attributed to dysrhythmias, cardiac failure, and cerebrovascular disease. The negative impact of thyrotoxicosis on cardiovascular morbidity and mortality was sustained even at 25 years after treatment for hyperthyroidism [10, 11].

The cardiovascular manifestations may not be reverted completely despite achievement of euthyroidism with treatment of hyperthyroidism. Despite our current understanding of the cardiovascular morbidity and mortality related to thyrotoxicosis, the characteristics and outcome of individual cardiac complication due to thyrotoxicosis have not been extensively studied. The only local data on thyrotoxicosis-related atrial fibrillation were reported by a single centre [12]. Among 160 hyperthyroid patients with atrial fibrillation, 74 (46%) were found to have persistent atrial fibrillation after one year of follow-up. Patients with persistent atrial fibrillation had a larger left atrial size, were less likely to be treated with beta-blockers, and had higher risk of ischemic stroke [12]. The same investigation group also recently reported a beneficial effect of anticoagulation (in terms of ischemic stroke free survival) for those who had persistent atrial fibrillation and a CHA_2_DS_2_-VASc score equal to or more than 1 in 642 patients who had concomitant hyperthyroidism and atrial fibrillation. On the contrary, the same effect was not observed in those who had self-limiting atrial fibrillation [13].

We therefore performed a retrospective study to examine the clinical characteristics and outcomes of thyrotoxic patients who presented atrial fibrillation. Possible factors associated with persistent atrial fibrillation despite restoration of euthyroidism were investigated. This may help to identify those who are at risk of persistent atrial fibrillation after control of thyrotoxicosis and anticoagulation therapy can then be initiated in due course.

## 2. Materials and Methods

This is a retrospective cohort study performed in the Department of Medicine and Geriatrics, Caritas Medical Centre, from 1 January 2004 to 30 June 2016. A search for all patients ≥ 18 years of age who had a registered diagnosis of hyperthyroidism or thyrotoxicosis and either one or more of the following cardiac manifestations was made with the Clinical Data Analysis and Reporting System (CDARS) from 1 January 2004 to 30 June 2016: atrial flutter, atrial fibrillation, paroxysmal atrial flutter, or paroxysmal atrial fibrillation. The hospital record of individual patient was retrieved to assess eligibility for inclusion.

Patients were included in the study if they had hyperthyroidism and concurrent atrial fibrillation and/or atrial flutter. Atrial fibrillation and atrial flutter were counted as a single entity in subsequent sections unless otherwise specified. Patients with preexisting heart failure, atrial fibrillation/atrial flutter, cardiomyopathy, significant mitral valve disease, congenital heart disease, amiodarone-induced thyrotoxicosis, and subclinical hyperthyroidism were excluded.

The record of every eligible patient was retrieved and reviewed. Demographic data including age, sex, body mass index, and smoking history were collected. History of hypertension, diabetes mellitus, ischemic heart disease, and ischemic stroke was noted. Hypertension was defined as systolic blood pressure (SBP) of ≥140 mmHg and/or diastolic blood pressure (DBP) of ≥90 mmHg, or if the patient was prescribed with antihypertensives. Diabetes mellitus (DM) was diagnosed according to the World Health Organisation (WHO) criteria [14, 15] or if the patient was prescribed with medications for DM. Ischemic heart disease was defined by the presence of a clinical diagnosis of coronary artery disease or myocardial infarction, positive results of a stress test, coronary computed tomography angiography, or invasive coronary angiography showing one or more vessels with stenosis of more than 50% [16, 17]. CHA_2_DS_2_-VASc was determined according to published guideline [18]. Thyroid function test results, cause of hyperthyroidism, anti-thyroid microsomal antibody titre and anti-thyroglobulin antibody titre, treatment modality of hyperthyroidism, and time to achieve euthyroidism were recorded. Data on cardiovascular assessment including blood pressure and heart rate on presentation, 12-lead surface electrocardiogram (ECG), presenting cardiac condition, echocardiographic findings, treatment, and the persistence of atrial fibrillation were adjudicated. Incident ischemic stroke, defined as a neurological deficit of sudden onset that persisted for more than 24 hours which corresponded to a vascular territory in the absences of primary hemorrhage and could not be explained by other causes at or after the onset of cardiac complications, was recorded [12, 19]. Associated clinical and echocardiographic characteristics for persistence of thyrotoxic atrial fibrillation were examined.

### 2.1. Assessment of Hyperthyroidism

Serum thyrotropin (TSH) level, free thyroxine (fT4) level, and free tri-iodothyronine (fT3) level were analyzed using commercially available immunoassays. Prior to 1 June 2010, the thyroid function tests were measured by the Abbott AxSym Immunoassay Analyzer. The reference range was 0.50–4.70 mIU/L for TSH, 9.1–23.8 pmol/L for fT4, and 2.2–5.3 pmol/L for fT3. The reported analytical coefficients of variability (CVs) ranged within 2.3–10.2% for TSH, 1.4– 6.5% for fT4, and 3.2–11.4% for fT3 [20]. Since 1 June 2010, thyroid function tests were measured with Beckman Coulter Access II Immunoassay Analyzer. The reference range was 0.17–4.37 mIU/L for TSH, 7.7–16.2 pmol/L for fT4, and 2.5–6.3 pmol/L for fT3. The analytic CVs of the assays ranged within 4.6–9.6% for TSH, 3.3–5.9% for fT4, and 4.8–6.6% for fT3, respectively. In-house correlation study between the two systems showed good correlation between the two analyzers for TSH (slope: 1.2065, *r* = 0.9985), fT4 (slope 1.054; *r* = 0.9899), and fT3 (slope: 0.679, *r* = 0.9899).

Anti-thyroid microsomal antibody (anti-TPO antibody) and anti-thyroglobulin antibody (anti-Tg antibody) titres were measured by commercial kits. Before 20 December 2011, anti-TPO antibody and anti-Tg antibody were measured by FujirebioSerodia agglutination assay and the cutoff points for positive values of both antibodies were >1 : 100 titre. Since 20 Dec 2011, the antibodies were measured by Inova Diagnostics enzyme-linked immunosorbent assay (ELISA) and the cutoff values for positivity of both antibodies were ≥101 units. Anti-TSH-receptor antibody was measured by Euroimmun ELISA kit and the cutoff value for positivity was ≥1 IU/l.

Hyperthyroidism was diagnosed in the presence of an elevated fT4 or fT3 level and a concomitant suppressed TSH level. Euthyroidism was defined as normal fT4 level and/or normal TSH level within the reference range. Graves' disease was diagnosed based on the presence of hyperthyroidism and at least one of the following: diffuse goiter on palpation or ultrasonography, positive tests for anti-TPO antibody or anti-Tg antibody, positive test for anti-TSH receptor antibody if the former was negative, homogenous increased uptake on pertechnetate thyroid scan, or presence of Graves' ophthalmopathy. Toxic multinodular goiter (MNG) was diagnosed based on clinical criteria, including the presence of hyperthyroidism, nodular goiter on palpitation or ultrasonography, and/or negative test for anti-thyroglobulin and anti-microsomal antibodies, and/or heterogenous uptake on pertechnetate thyroid scan. Toxic adenoma was diagnosed in the presence of solitary uptake with suppression of surrounding thyroid tissue on pertechnetate thyroid scan.

### 2.2. Cardiac Assessment

Diagnosis of atrial fibrillation/flutter was confirmed with standard 12-lead electrocardiogram. Sinus conversion was confirmed with standard 12-lead electrocardiogram during hospital admission or at outpatient follow-up. Persistence of atrial fibrillation/atrial flutter was defined as failure of spontaneous sinus conversion at 12 months after achievement of euthyroidism. Heart failure was diagnosed by the modified Framingham criteria which have 100% sensitivity and 78% specificity for the diagnosis of heart failure [13, 16, 21]. These criteria were classified as major or minor. The major criteria were paroxysmal nocturnal dyspnoea, orthopnoea, jugular venous distention, pulmonary rales, cardiomegaly, pulmonary edema, presence of a third heart sound, and weight loss of 4.5 kilograms in 5 days in response to diuretic therapy. The minor criteria were edema, nocturnal cough, dyspnoea on ordinary exertion, hepatomegaly, pleural effusion, and tachycardia of more than 120 beats per minute. A patient was considered to have heart failure if two major criteria or if one major and two minor criteria were present concurrently.

Two-dimensional and M-mode transthoracic echocardiography (TTE) was performed by the cardiology team using the Vivid 7-Dimension machine with a 3.5 MHz transducer according to the recommendations of the American Society of Echocardiography (ASE) [22, 23]. Left ventricular (LV) systolic dysfunction was defined as left ventricular ejection fraction (LVEF) < 50% by M-mode measurement. Valvular regurgitation was classified as “absent,” “mild,” “moderate,” or “severe” using a semiquantitative method [24]. Pulmonary arterial systolic pressure (PASP) was calculated by estimating the systolic pressure gradient between the right ventricle and right atrium with continuous Doppler interrogation of the tricuspid regurgitation jet and then adding the mean right atrial pressure which was estimated to be 5 mmHg [25, 26]. Pulmonary hypertension was defined as pulmonary arterial systolic pressure of at least 35 mmHg. Dilated cardiomyopathy was defined by the presence of heart failure together with left ventricular dilatation (a left ventricle end diastolic diameter of >117% predicted value corrected for age and body surface area) and LVEF < 45% on TTE [27, 28]. Isolated right heart failure was diagnosed with clinical evidence of peripheral fluid retention and echocardiographic findings of dilated right ventricle, elevated pulmonary arterial systolic pressure, and moderate to severe tricuspid regurgitation in the absence of LV systolic dysfunction [29, 30].

### 2.3. Statistical Analysis

All analyses were computed using the Statistic Package for Social Science (SPSS for windows, version 19.0, SPSS Inc., Chicago, IL USA). Shapiro-Wilk test was used to check for normality for continuous variable. Data were shown as absolute number and percentages for categorical variables and mean ± standard deviation (SD) or median and interquartile range (IQR) for continuous variables as appropriate. Comparisons between two independent groups were undertaken using two-tailed Fisher exact test or Chi-square test for categorical variables and independent *t*-test or Mann–Whitney *U* test for continuous variables. Changes in continuous variables at baseline and follow-up were made by paired *t*-test for continuous variables. Kaplan Meier analysis and log-rank test were used to compare rates of incident ischemic stroke in patients with hyperthyroidism-induced atrial fibrillation. Variables that were found to be significant on univariate analysis were put into a logistic regression model for multivariate analysis. Strength of association was quantified with adjusted odds ratios (OR) and 95% confidence intervals (95% CI). A two-tailed *p* value of less than 0.05 was considered statistically significant.

### 2.4. Ethics Committee Approval

The study was approved by the Local Research Ethics Committee (Approval Number: KWC REC 77-13).

## 3. Results

### 3.1. Baseline Characteristics of Subjects

During the 12.5 years' period from 1 January 2004 to 30 June 2016, a total of 1918 patients had a registered diagnosis of thyrotoxicosis via CDARS search in the Department of Medicine and Geriatrics in Caritas Medical Centre. Among these patients, 133 (6.9%) patients presented with atrial fibrillation, while 63 (3.3%) patients presented with coexisting atrial fibrillation and heart failure. Of the 106 patients who had a baseline echocardiographic examination, measurement of tricuspid regurgitation was documented in 104 (98.1%) patients and 54 (51.2%) of them had pulmonary hypertension.

The mean age of the cohort was 63 ± 15 years (range 28–90 years). Seventy-five (56.4%) patients were female. The etiology of hyperthyroidism was Graves' disease in 70 (52.6%) patients, toxic multinodular goiter in 50 (37.6%) patients, toxic adenoma in 2 (1.5%) patients, and indeterminate in 11 (8.3%) patients. Median TSH was 0.03 mIU/l (IQR 0.01–0.03 mIU//l) and mean fT4 was 2.2 ± 0.99 times upper limit of normal (mean fT4 was 46 ± 18 pmol/L prior to 1 June 2010 and 42 ± 20 pmol/L on or after 1 June 2010). Median time to euthyroidism was 1.6 months (IQR 0.8–3.2 months) after treatment.

### 3.2. Outcome of Atrial Fibrillation

**Figure 1:**
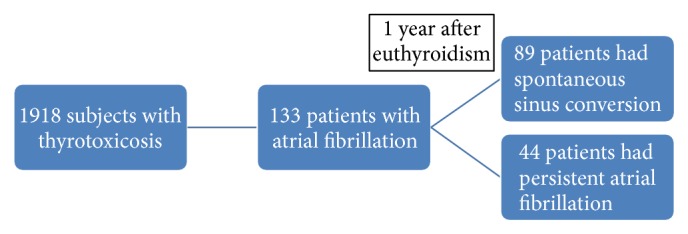
Subjects included in the study.

Over a median follow-up period of 47 months (IQR 24–82 months), 44 (33.1%) patients had persistent atrial fibrillation and 89 (66.9%) patients had spontaneous conversion to sinus rhythm (Figure 1). The baseline demographic data of the two groups of patients are shown in Table 1.

**Figure 2:**
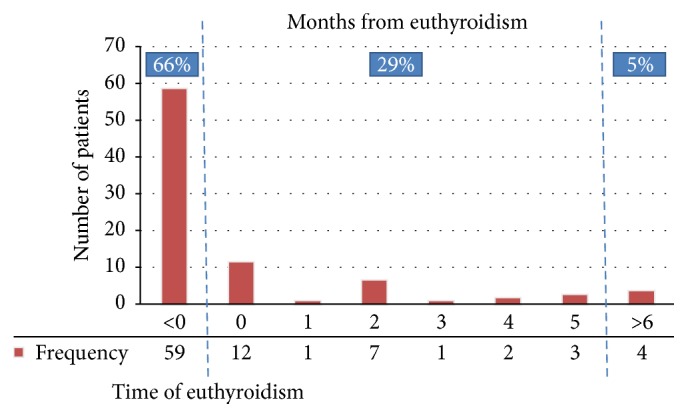
Time of spontaneous sinus conversion.

In patients with spontaneous sinus conversion, the time to sinus conversion ranged from 0 to 580 days since diagnosis with a median time of 6 days (IQR 2–90 days). In other words, the time of reversal ranged from 184 days before euthyroidism to 355 days after euthyroidism (median 27 days before euthyroidism; IQR 63 days before euthyroidism and 7 days after euthyroidism). About 66% (59 patients) had spontaneous sinus conversion before attaining euthyroidism, and another 30% (26 patients) did so within 6 months of having achieved a euthyroid state. Only 4 (3%) patients had spontaneous sinus conversion after euthyroidism for more than 6 months (Figure 2). No spontaneous reversal was observed later than 355 days after achieving euthyroidism.

In univariate analysis, patients with persistent atrial fibrillation were more likely to have history of smoking (65.9% versus. 42.3%, *p* = 0.019), have a lower free T4 level (mean fT4 x ULN = 1.93 ± 0.86 versus. 2.33 ± 1.03, *p* = 0.033), have a past episode of thyrotoxicosis (40.9% versus. 23.6%, *p* = 0.039), and be treated with digitalis (43.2% versus. 20.2%, *p* = 0.005) and angiotensin-converting-enzyme inhibitors (ACEI)/angiotensin II receptor blockers (ARB) (43.2% versus. 20.2%, *p* = 0.045) compared with those who had spontaneous sinus conversion (Table 1).

Echocardiographic examination was performed in 109 patients when they were still in the toxic state, including 68 patients with spontaneous sinus conversion and 41 patients with persistent atrial fibrillation (Table 2). The echocardiographic findings are shown in Table 2. Mean LA diameter (3.89 ± 0.75 cm versus. 3.41 ± 0.70 cm, *p* = 0.003) and LVDD (4.74 ± 0.80 cm versus. 4.41 ± 0.69 cm, *p* = 0.030) were significantly larger in patients with persistent atrial fibrillation than in those with spontaneous reversal of atrial fibrillation. Moderate to severe tricuspid regurgitation was more common in patients with persistent atrial fibrillation (28.9% versus 15.4%, *p* = 0.099) though the difference did not reach statistical significance, while PASP between the two groups was not significantly different.

Multivariate logistic regression adjusted for variables found to be significant on univariate analysis was performed. Treatment with digitalis and ACEI/ARB was excluded as this was unlikely a causal factor. History of smoking (adjusted OR 4.9, 95% CI [1.8,14], *p* = 0.002), a larger left atrial diameter (adjusted odd ratio 2.6, 95% CI [1.2,5.5], *p* = 0.014), and a relatively lower free thyroxine level at diagnosis (adjusted odd ratio 2.1, 95% CI [1.2,3.5], *p* = 0.008) were associated with persistence of atrial fibrillation on multivariate analysis (Table 3).

**Figure 3:**
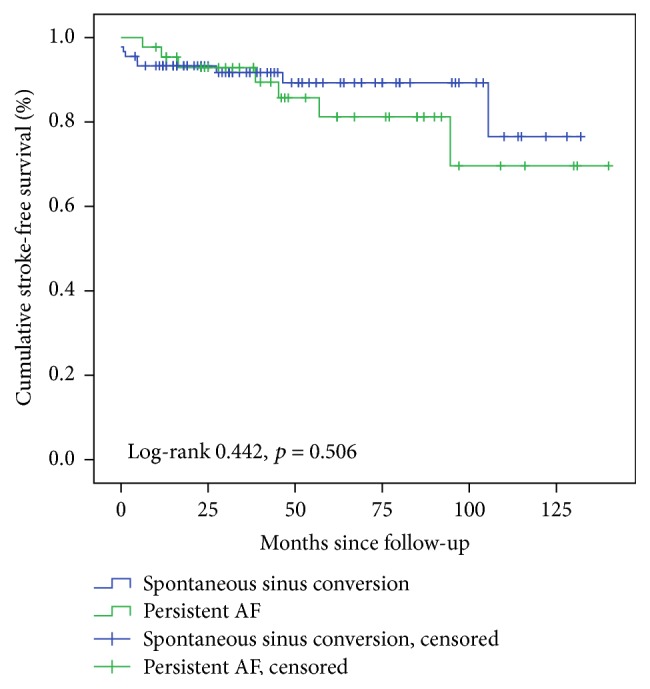
Stroke free survival in patients with thyrotoxic atrial fibrillation.

Ischemic stroke occurred in 16 (12.0%) patients who had thyrotoxic atrial fibrillation during the study period. The annualized rate of ischemic stroke was 3.1%. The rate of stroke was numerically higher among patients who had persistent atrial fibrillation than those with spontaneous sinus conversion though the difference was not statistically significant (15.9% versus 10.1%; log-rank 0.442, *p* = 0.506) (Figure 3). Patients who sustained an ischemic stroke were older (71 ± 11 years versus. 62 ± 16 years, *p* = 0.023). There was a trend towards higher mean CHA_2_DS_2_VASc score (2.9 [1.7] versus 2.3 [1.7], *p* = 0.153) among those who sustained an ischemic stroke but the difference was not statistically significant. There was no significant difference in the rates of ischemic stroke occurring in those taking warfarin or direct oral anticoagulant or those not taking it (16.7% versus. 11.6%, *p* = 0.638), but the number of patients on anticoagulation was small (*n* = 12, 9% of total cohort) (Table 4).

## 4. Discussion

The cardiovascular effects of hyperthyroidism are characterized by increased preload with low systemic vascular resistance, a high heart rate, and increased myocardial consumption [31]. Hyperthyroidism has myriad cardiovascular manifestations and common clinical manifestations include palpitation, exertional dyspnoea, reduced exercise capacity, sinus tachycardia, and systolic hypertension [32]. Specific cardiovascular complications that are commonly encountered include atrial fibrillation and heart failure. With the advent of more advanced cardiovascular tests, diastolic dysfunction [33], pulmonary hypertension [34], and dilated cardiomyopathy [35, 36] have been more recently recognised as a direct consequence due to thyrotoxicosis. Ventricular arrhythmias have been reported in isolated reports but larger systemic studies have not confirmed such an association [32, 37, 38].

### 4.1. Epidemiology of Thyrotoxic Atrial Fibrillation

Atrial fibrillation is one of the most common cardiovascular manifestations of hyperthyroidism. It occurs in 2% to 20% of newly diagnosed hyperthyroid patients, compared to 0.5–9.0% of the general population [4, 6, 31, 39, 40]. Hyperthyroidism is associated with shortened action potential duration, increased left atrial pressure, and enhanced atrial automaticity which contribute to the development of atrial fibrillation [41]. Our study showed that 133 (6.9%) patients in a cohort of 1918 hyperthyroid patients had atrial fibrillation. By contrast, Petersen and Hansen reported prevalence rate of 14.9% (91 out of 610 patients) of atrial fibrillation in subjects with thyrotoxicosis [42] while Iwasaki et al. reported that 19 (21.4%) out of 92 patients with Graves' disease had atrial fibrillation [43]. These prevalence rates were based on historical cohorts decades ago. On the other hand, our finding was in closer approximation to the data from more contemporary cohorts. In a British prospective study evaluating the prevalence of cardiovascular abnormalities in 392 patients with overt hyperthyroidism [32], atrial fibrillation was present in 29 (7.2%) patients at recruitment. In a nationwide case control study in Denmark, 40628 patients were identified to have hyperthyroidism based on hospital discharge coding and 3362 (8.3%) were found to have atrial fibrillation [44]. The apparent decreased prevalence was attributed to earlier diagnosis and more precise treatment by means of more sensitive thyroid hormone assays [39].

### 4.2. Persistent Atrial Fibrillation and Associated Factors

Persistent atrial fibrillation occurred in approximately 35–50% of patients despite control of hyperthyroidism [12, 39, 45, 46]. Data from a Japanese institute suggested that approximately 1.7% of patients with newly diagnosed hyperthyroidism would develop persistent atrial fibrillation [39]. In the present study, it was observed that 44 (2.3%) out of a total cohort of 1918 patients had persistent atrial fibrillation. Alternatively, in those 133 patients with thyrotoxicosis-related atrial fibrillation, 33.1% had persistent atrial fibrillation. This rate was found to be intermediate between those observed in two previous studies in the Chinese population. Siu et al. [12] prospectively studied 160 hyperthyroid patients who had atrial fibrillation and they found that, at one year of follow-up, 74 (46%) patients remained in atrial fibrillation. Zhou et al. [46] reported the outcome of 83 hyperthyroid patients with atrial fibrillation who had undergone radioactive iodine treatment. At up to 5 years of follow-up, persistent atrial fibrillation was observed in 27 (32.5%) patients. In Japan, Nakazawa et al. [45] reported persistent atrial fibrillation in 62 out of 163 (38%) patients during a mean follow-up of 34 months.

#### 4.2.1. Left Atrial Diameter

LA dilatation is a hallmark of LA structural remodeling and is linked to atrial fibrosis and subsequent atrial fibrillation [47]. An enlarged LA diameter has been linked to increased risk of incident atrial fibrillation in nonthyrotoxic subjects [48]. The present study showed that a larger LA diameter was associated with persistent atrial fibrillation. Similarly, patients with persistent atrial fibrillation at 1 year were also found to have a larger baseline LA diameter compared to those with spontaneous sinus conversion (3.6 ± 0.1 cm versus 3.9 ± 0.1 cm, *p* = 0.01) by Siu et al. [12]. The association remained significant (*p* = 0.02) upon multivariate regression analysis. Unlike in nonthyrotoxic patients whose LA size might predict persistence of atrial fibrillation after cardioversion, an enlarged LA did not seem to pose a thyrotoxic patient at risk of recurrence of atrial fibrillation after cardioversion [49, 50]. An enlarged LA diameter may predict a subset of patients with thyrotoxic atrial fibrillation who can benefit most from timely cardioversion as they are less likely to revert spontaneously to sinus rhythm, yet at the same time have a higher maintenance rate compared with nonthyrotoxic patients. The higher maintenance rate is attributed to the reversible nature of the effect of hyperthyroidism on atrial effective refractory period upon treatment [2].

#### 4.2.2. History of Smoking

History of smoking was associated with persistent atrial fibrillation in the present study. In nonthyrotoxic subjects, ever smokers were found to have increased incident risk of atrial fibrillation in large prospective population-based studies [51, 52]. Putative mechanisms include oxidative stress, inflammation, atrial fibrosis, and nicotine stimulating sympathetic neurotransmission. The association of smoking with thyrotoxic atrial fibrillation was less clearly defined. Only in one study the relationship between smoking and persistent thyrotoxic atrial fibrillation was examined and no association was observed [12]. This might be attributed to the smaller number of patients with smoking history in their study (41 out of 160, 26%) compared to the present study (68 out of 133, 51%) and any observed difference in exposure was more likely to be mitigated.

#### 4.2.3. Tricuspid Regurgitation

A number of cases with moderate and severe tricuspid regurgitation and isolated right heart failure with marked right heart dilatation have been reported in thyrotoxic subjects in the literature, and majority of them had coexisting atrial fibrillation [29, 30, 53–57]. Mercé et al. prospectively studied the echocardiographic features of 39 hyperthyroid patients over a mean follow-up of 14 months [58]. They found that patients with moderate and severe tricuspid regurgitation were more likely to have atrial fibrillation (86% [6/7] versus 3% [1/32], *p* = 0.01). Chronic atrial fibrillation predisposes the heart to progressive right atrial and ventricular dilatation and functional tricuspid regurgitation, and severe tricuspid regurgitation was considered a marker of right heart failure in nonthyrotoxic subjects [59]. Our study also showed that thyrotoxic patients with persistent atrial fibrillation had a trend towards larger mean RV diameter (2.54 ± 0.83 cm versus. 2.26 ± 0.69 cm, *p* = 0.140) and higher rate of moderate and severe tricuspid regurgitation (*p* = 0.099). This was consistent with the findings in nonthyrotoxic subjects, suggesting thyrotoxicosis as a cause for right heart failure.

#### 4.2.4. Heart Failure

Back in the 1980s, Nakazawa et al. [45] had found that 20% (20 out of 101) of patients with spontaneous sinus conversion had heart failure in comparison to 42% (26 out of 62) of patients with persistent atrial fibrillation (*p* < 0.01). More recently, Zhou et al. [46] found that thyrotoxic subjects with persistent atrial fibrillation had lower LVEF (40% versus 45%, *p* < 0.05) than those who had spontaneous sinus conversion. In line with the published literature, the present study also showed a trend (*p* = 0.057) towards higher prevalence of heart failure among subjects with persistent atrial fibrillation and its related treatment (e.g., digitalis and ACEI/ARB). Heart failure had also been shown to be a risk factor of permanent atrial fibrillation in nonthyrotoxic subjects [60]. Presence of heart failure usually indicates more advanced cardiac insult and cardiac remodeling may become irreversible with prolonged exposure of excess thyroid hormone [61].

Nevertheless, a lower mean LVEF was not observed in patients with persistent atrial fibrillation (mean LVEF 56%) as compared to those with spontaneous sinus conversion (mean LVEF 59%) in the present study despite a high rate of heart failure. Some of these patients might have diastolic dysfunction which had not been rigorously studied in the present study. Diastolic dysfunction had been associated with increased risk of atrial fibrillation [62, 63] and high prevalence of diastolic dysfunction had been observed in subjects with thyrotoxicosis in sinus rhythm especially in older subjects. Goland et al. [35] reported that almost one-third (22 out of 70 patients) of hyperthyroid patients in sinus rhythm had diastolic dysfunction. In patients younger than 40 years old, the rate was 17.9% while it rose to 100% in those older than 60 years old. Systemic study on diastolic function in patients with thyrotoxic atrial fibrillation has not been reported in the literature so far.

#### 4.2.5. Past Episode of Thyrotoxicosis

Having a past episode of thyrotoxicosis was also found to be linked to persistent atrial fibrillation in our study. This factor has been less examined in published literature. Nakazawa et al. [45] excluded subjects with recurrence of hyperthyroidism in their cohort. Another study did not look into such an association [12]. In theory, a previous episode of thyrotoxicosis might expose a patient to a longer duration of excess thyroid hormone exposure. This may result in cardiac remodeling and arrhythmogenesis that is less reversible. In line with this, Zhou et al. [46] found that a longer duration of hyperthyroidism more than 5 years prior to radioactive iodine treatment was associated with a higher change of persistent atrial fibrillation (relative risk 3.08, 95% CI: 1.2–11.4, *p* < 0.01).

#### 4.2.6. Free Thyroxine Level

Data from out study showed that relatively lower level of free T4 was associated with persistent atrial fibrillation after multivariable adjustment. It was an unexpected finding but a similar observation has been reported by Siu et al. on thyrotoxic heart failure [64]. In their analysis of 34 patients who had concomitant hyperthyroidism and heart failure, 16 (47%) had left ventricular (LV) dysfunction as determined by LVEF < 50%. A lower free T4 level was observed among these patients as compared to those without LV dysfunction (fT4 level: 39 ± 4 pmol/L versus. 66 ± 12 pmol/L, *p* = 0.04). The exact reason was unclear but may be related to a more prolonged duration of “subclinical” hyperthyroidism as seen in elderly patients with multinodular goiter [64].

### 4.3. Ischemic Stroke and Thyrotoxic Atrial Fibrillation

In the present study, 16 out of 133 patients (12.0%) with thyrotoxic atrial fibrillation had an ischemic stroke over a median follow-up of 47 months. This was in agreement with several recent prospective observational studies which reported an incidence of about 8–10% of ischemic stroke in patients with hyperthyroidism-induced atrial fibrillation as compared to an incidence of about 3-4% of ischemic stroke in sex and age matched controls [12, 13, 65, 66]. Moreover, hyperthyroid patients with atrial fibrillation were at higher risk of stroke than patients with nonthyroid atrial fibrillation in two previous studies by Siu et al. [12] and Chen et al. [65], although a more recent study by Chan et al. did not show such an observation [13].

Of note, old age has been reported as the only factor, or one of the two factors (age > 75 and renal failure) associated with ischemic stroke in two previous studies where traditional risk stratification scheme (e.g., CHADS2 or CHA2DS2VASc score) was less discerning [13, 67]. The finding has been replicated in our study although our study is not adequately powered to test for the multiple risk categories.

Subjects with persistent atrial fibrillation were also at higher risk of ischemic stroke by Siu et al. (hazard ratio 13.0; 95% CI 2.88, 58.8, *p* < 0.01) compared to those who had sinus conversion [12]. Treatment with warfarin has been shown to be associated with lower risk of ischemic stroke in Chinese subjects with hyperthyroid atrial fibrillation [12, 65] and the benefit may be more pronounced in those with persistent atrial fibrillation with additional risk factors [13] although evidence from an adequately powered randomized controlled trial is lacking [68]. Nevertheless, it remains highly probable that persistent atrial fibrillation related to hyperthyroidism is linked to higher risk of ischemic stroke, especially in the presence of some high risk features (e.g., older age), and further prospective studies are warranted to investigate the risk-benefit ratio of anticoagulation therapy in thyrotoxic atrial fibrillation.

### 4.4. Limitations

Our study was one of the few studies which provided detailed analysis of persistent thyrotoxic atrial fibrillation in a relatively large group of patients over a long follow-up period. There was also very high retention and follow-up rate among these subjects, probably related to their cardiac complications.

There were several limitations and drawbacks in our study. Firstly, baseline echocardiographic examination was not performed in all patients and some parameters (mainly RV diameter and PASP) were missing. Secondly, echocardiogram was performed by different ultrasonographers over the study period and thus interobserver variation cannot be excluded. Nevertheless, measurements of LV systolic function from M-mode have proven to be reproducible with low intraobserver and interobserver variability [23]. Thirdly, the number of incident ischemic strokes was small which has significantly weakened the statistical power of our association analysis. Last but not least, the retrospective nature of the present study precluded assessment of the causal relationship between the clinical factors of interest and the cardiac conditions being examined.

## 5. Conclusion

In conclusion, atrial fibrillation occurred in 6.9% of thyrotoxic patients in the present study and about one-third of them had persistent atrial fibrillation over a median follow-up of 4 years despite control of hyperthyroidism. High rate of stroke was also observed among those who had thyrotoxic atrial fibrillation in the present study especially in older patients. History of smoking, a dilated LA diameter, and a relatively lower free T4 level were independently associated with persistent atrial fibrillation. Patients with the above clinical features should be carefully monitored for the persistence of arrhythmia for which anticoagulation therapy should be considered, given the higher reported risk of ischemic stroke and observed benefit of anticoagulation in patients with hyperthyroidism-induced atrial fibrillation in other studies. A randomized controlled trial to examine the effect of anticoagulation therapy on preventing ischemic stroke in thyrotoxic atrial fibrillation is eagerly needed.

## Figures and Tables

**Table 1:** Comparison of clinical characteristics of patients with spontaneous reversal of atrial fibrillation to sinus rhythm versus patients with persistent atrial fibrillation.

	Spontaneous reversal	Persistent atrial fibrillation	*p* value
*n*	89	44	
Age (years)	64 (15)	63 (16)	0.893
Sex (female)	55 (61.8%)	20 (45.5%)	0.074
History of smoking	39 (42.3%)	29 (65.9%)	***0.019*** ^*∗*^
BMI (kg/m2)	22.1 (3.7)	22.1 (3.9)	0.938
Systolic BP (mmHg)	148 (26)	144 (24)	0.374
Diastolic BP (mmHg)	82 (18)	80 (16)	0.659
Heart rate on 1st ECG (bpm)	132 (32)	128 (27)	0.521
DM	14 (15.7%)	9 (20.5%)	0.498
HT	31 (34.8%)	17 (38.6%)	0.667
IHD	7 (7.9%)	1 (2.3%)	0.270
Median months (IQR) of follow-up	40 (19–71)	48 (26–87)	***0.006*** ^*∗*^
Etiology of thyrotoxicosis			
(i) Graves' disease	43 (48.3%)	27 (61.4%)	0.347
(ii) Multinodular goitre	37 (41.6%)	13 (29.5%)	
(iii) Others/indeterminate	9 (10.1%)	4 (9.1%)	
fT4 (xULN)	2.33 (1.03)	1.93 (0.86)	***0.033*** ^*∗*^
fT4 (pmol/l)			
(i) Before 6/2010	49.0 (19.4) (*n* = 47)	40.8 (15.0) (*n* = 25)	0.064
(ii) After 6/2010	42.5 (19.0) (*n* = 42)	36.2 (17.6) (*n* = 19)	0.233
Positive anti-TPO antibody	37 (54.4%) (*n* = 68)	19 (65.5) (*n* = 29)	0.311
Positive anti-Tg antibody	25 (36.2%) (*n* = 69)	15 (51.7%) (*n* = 29)	0.154
Heart failure	37 (41.6%)	26 (59.1%)	0.057
Dilated cardiomyopathy	3 (3.4%)	5 (11.4%)	0.115
Having a past episode of thyrotoxicosis	21 (23.6%)	18 (40.9%)	***0.039*** ^*∗*^
Median time to achieve euthyroidism (months, IQR)	2 (1–3)	2 (1–3)	0.512
Radioactive iodine as treatment for thyrotoxicosis	35 (39.3%)	21 (47.7%)	0.356
Beta blockers	54 (60.7%)	23 (52.3%)	0.356
ACEI/ARB	18 (20.2%)	16 (36.4%)	***0.045*** ^*∗*^
Digitalis	18 (20.2%)	19 (43.2%)	***0.005*** ^*∗*^
Aspirin	31 (34.8%)	18 (40.9%)	0.494
Warfarin	6 (6.7%)	6 (13.6%)	0.192
Incident ischaemic stroke	9 (10.1%)	7 (15.9%)	0.506

*Note*. bpm: beats per minute; BMI: body mass index; BP: blood pressure; DM: diabetes mellitus; IHD: ischemic heart disease; ULN: upper limit of normal; ACEI: angiotensin converting enzyme inhibitor; ARB: angiotensin II receptor blocker. Values are expressed as *n* (%) or mean (SD) unless otherwise specified; ^*∗*^*p* < 0.05.

**Table 2:** Comparison of the echocardiographic findings in patients with spontaneous reversal of atrial fibrillation to sinus rhythm and patients with persistent atrial fibrillation.

	Spontaneous reversal (group 1)	Persistent atrial fibrillation (group 2)	*p* value
*n* ^#^	68	41	
LVEF (%)	59 (13)	57 (14)	0.478
LVSD (cm)	3.06 (0.70)	3.33 (0.92)	0.107
LVDD (cm)	4.41 (0.69)	4.74 (0.80)	***0.030*** ^*∗*^
LA diameter (cm)	3.41 (0.70)	3.89 (0.75)	***0.003*** ^*∗*^
RV diameter (cm)	2.26 (0.69)	2.54 (0.83)	0.140
RVSP (mmHg)	39 (9)	41 (10)	0.251
MR			
(i) Absent/mild	56 (86.2%)	32 (82.1%)	0.575
(ii) Moderate/severe	9 (13.8%)	7 (17.9%)	
TR			
(i) Absent/mild	55 (84.6%)	27 (71.1%)	0.099
(ii) Moderate/severe	10 (15.4%)	11 (28.9%)	

*Note*. LVEF: left ventricular ejection fraction; LVSD: left ventricular end systolic diameter; LVDD: left ventricular end diastolic diameter; LA: left atrium; RV: right ventricle; PASP: pulmonary artery systolic pressure; MR: mitral regurgitation; TR: tricuspid regurgitation. Values are in *n* (%) or mean (SD). ^#^Data were present for 88–100% of parameters except RV diameter (59–63%, *n* = 65 for total cohort, *n* = 39 for group (1), and *n* = 26 for group (2)) and PASP (68–77%, *n* = 79 for total cohort, *n* = 51 for group (1), and *n* = 28 for group (2)); ^*∗*^*p* < 0.05.

**Table 3:** Multivariate analysis to determine factors associated with persistent atrial fibrillation.

Factor	Adjusted odd ratios	95% CI	*p* value
Current/past smoker	4.9	1.8, 14	***0.002*** ^*∗*^
Lower free T4 at diagnosis	2.1	1.2, 3.5	***0.008*** ^*∗*^
Larger LA diameter	2.6	1.2, 5.5	***0.014*** ^*∗*^
Having a past episode of thyrotoxicosis	1.8	0.29, 2.1	0.642
LVDD	1.1	0.49, 2.2	0.925

*Note*. LVDD: left ventricular end diastolic diameter; LA: left atrium; ^*∗*^*p* < 0.05.

**Table 4:** Comparison of clinical characteristics in patients who did not have a stroke and patients who had sustained an ischemic stroke.

	No stroke	Ischemic stroke	*p* value
*n*	117	16	
Age	62 (16)	71 (11)	***0.023*** ^*∗*^
CHA_2_DS_2_-VASc score	2.3 (1.7)	2.9 (1.7)	0.185
FT4 (xULN)	2.22 (1.00)	2.00 (0.94)	0.407
Persistent AF	37 (31.6%)	7 (43.8%)	0.327
LA size	3.59 (0.82) (*n* = 91)	3.41 (0.71) (*n* = 13)	0.453
Warfarin or DOAC	10 (8.5%)	2 (12.5%)	0.638

Note: *n*: number of subjects; fT4: free thyroxine level; AF: atrial fibrillation; LA: left atrial; DOAC: direct oral anticoagulant. Values are in *n* (%) or mean (SD); ^*∗*^*p* < 0.05.
